# Knowledge, attitude and practices of farmers and experts about the effects of pesticide residues on agricultural product users and ecosystems: A case of Fogera District, Ethiopia

**DOI:** 10.1371/journal.pone.0292838

**Published:** 2023-12-07

**Authors:** Abebaw Abaineh, Dessalegn Ejigu, Minaleshewa Atlabachew, Eshete Dejen, Gashaw Tilahun

**Affiliations:** 1 Department of Fisheries and Aquatic Sciences, College of Agricultural and Environmental Sciences, Bahir Dar University, Bahir Dar, Ethiopia; 2 Department of Biology, College of Science, Bahir Dar University, Bahir Dar, Ethiopia; 3 Department of Chemistry, College of Science, Bahir Dar University, Bahir Dar, Ethiopia; 4 PM, Environment Protection, Agriculture & Environment Division, IGAD Secretariat, Djibouti, Republic of Djibouti; Wollo University, ETHIOPIA

## Abstract

Pesticides are chemicals used to control different types of pests. Though pesticides played a role in improving the quantity and quality of production, they have been threatening ecosystems and posed effects on humans in different parts of the world. Unfortunately, there were no studies made about the effects of pesticide residues on ecosystems and consumers in the Fogera District of Amhara Region, Ethiopia. Hence, the main objective of this study was to understand the knowledge, attitude, and practices of respondents about the effects of pesticide residues on ecosystems and consumers. A cross-sectional survey complemented by focus group discussions and field observations was used to gather the required data for the study. The close-ended data were analyzed using descriptive statistics, logistic regressions, and independent t-test, and data from open-ended questions were grouped and summarized based on their similarities. The findings of the study confirmed that there was significant knowledge, attitude, and practices difference between farmers and consumers about the effects of pesticide residues on ecosystems and humans. Farmers used highly toxic pesticides to control pests and improve the glossiness of vegetables and khat. Though they didn’t use the sprayed vegetables for their home consumption, some of the farmers deliberately supplied pesticide-sprayed vegetables without worrying about the negative effects of the pesticides on the consumers. There were also fishing practices from rivers after intoxicating the fish using the pesticide sprayed feed. This, in turn, might poison individuals who consume the fish. In general, pesticide application practices and consumption of pesticide-sprayed foodstuffs and surface water might pose serious health risks to ecosystems and humans. To minimize the negative effects of pesticides, rigorous awareness-raising on the effects and management of pesticides, enforcement of laws, delineation of the pesticide free buffer zone for waters, the establishment of a clear pesticide supply chain to the end users, ecosystem assessment and food safety monitoring schemes are highly required.

## 1. Introduction

Pesticides are chemicals used to prevent, eradicate, or control pests that affect food, agricultural products, wood and wood products, and animal feedstuffs [1–5]. Domesticated animals might also receive these pesticides for the management of insects, arachnids, and other pests in or on their bodies [6]. They are widely utilized in the developing world, and their demand is rising as a result of the current crop production system, which places a premium on high agricultural yields [2, 7]. Pesticides enable farmers to avoid up to 78%, 54%, and 32% losses of fruits, vegetables, and cereals, respectively [5]. They are relatively easy to apply, cost-effective, and sometimes the only option to control pests [8, 9].

Pesticides are a threat to all ecosystem components, including humans, animals, and insects [7, 10]. Globally, agricultural practices are the major sources of water pollution that discharge huge amounts of organic and inorganic pollutants into water bodies [11]. A rise in agricultural productivity is accompanied by an increase in pesticide use per unit area and magnified the concentration of pesticide residues in foodstuff and the environment to the level that affects the health of humans and other living organisms [12, 13]. In Ethiopia, pesticides have been used intensively in agricultural sectors without worrying about their negative environmental and human effects [14]. Thus, pesticide use is the most common practice in the Amhara Region of Ethiopia to control pests, diseases, and weeds [15]. More than 50% of pesticides registered and used in the country, including the study area, are on the list of highly hazardous pesticides (HHPs) [16], and they might cause adverse negative effects on the environment and humans [17].

Lake Tana Biosphere Reserve is one of the biodiversity hotspot areas of the Amhara Region [18], and agriculture is the most dominant and important economic activity of the Lake Tana Watershed, including Fogera District, where Livestock production and cultivation are the leading economic activities [19]. Most wetlands and catchment areas of Lake Tana have progressively been transformed from vegetated to cultivated land in the last 28 years [20]. Recently, the land use/land cover change in the Lake Tana Watershed from vegetated and grazing land to cultivation, settlement, and investment has been intense and ever-increasing [21].

There were different studies conducted on the attitudes and practices of farmers regarding pesticide use and handling in northwestern Ethiopia, particularly on the Lake Tana Watershed [22, 23]. However, previous studies mainly focused on farmers’ pesticide management practices during application in the field and during storing against prescribed labelings of pesticides to minimize their side effects [23, 24]. There were no studies made about the knowledge, attitude, and practices (KAPs) of farmers and consumers about the effects of pesticide residues on aquatic ecosystems and consumers of agricultural products. Therefore, this study focused on the effects of pesticide residues on human and ecosystem health in Fogera District, northwestern Ethiopia. Hence, the main objective of this study was to evaluate KAPs of farmers and experts regarding the effects of pesticide residues on humans and ecosystems.

## 2. Materials and methods

### 2.1. Description of the study area

**Fig 1:**
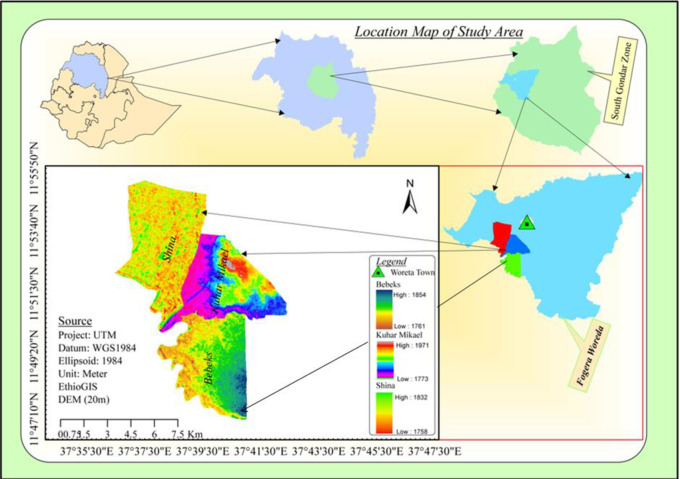
Location map of Fogera District. Source: BORIS DOI:10.7892/boris.67058 [28].

The study was conducted in the Fogera District of the South Gondar Administrative Zone, northwestern Ethiopia (Fig 1). Fogera District is one of the thirteen Districts in the South Gondar Zone, and it is bordered by Dera District on the South, Lake Tana on the West, Rib River on the North, which separates it from Libo-Kemekem District, Ebenat District on the northeast, and Farta District on the East [25, 26]. Woreta, the capital of the District, is located 625 km northwest of Addis Ababa and 55 km from the Regional capital, Bahir Dar [27]. The District is situated at 11°46’ to 11°59’N latitudes and 37°33’ to 37° 52’ E longitudes [27], and its altitude ranges from 1774 to 2415m a.s.l. [26]. The study area has an annual rainfall ranging from 1103 to 1336 mm [25, 27]. Fogera District is one of the Districts that share the Fogera floodplain in the region, and the floodplain areas in the District are waterlogged for more than 4 months, starting from July of each year. Fogera District Environment and forest protection office has given permission to the researcher to undertake the KAPs study in the selected sites of the District.

### 2.2. Data collection methods

A cross-sectional survey complemented with Focused Group Discussions (FGD) and field surveys was used to evaluate the KAPs of the respondents. The study was conducted between January and March 2022. Qualitative and quantitative data were gathered on KAPs of respondents about the effects of pesticide residues on human and ecosystems’ health. Three rural sub-districts of Fogera District that practice intensive irrigation agriculture, such as Shina, Bebekis, and Kuhar-Michael, were purposively selected for data collection. The sampled sub-districts were among the most frequently cultivated areas of the District. It was assumed that increased cultivation frequency increased the frequency of pesticide use per unit area per year [13].

A reconnaissance survey was conducted prior to the actual data collection to assess the worst scenario about the effects of pesticide residues on ecosystems and consumers of agricultural products. Furthermore, the sites were easily accessible and cost-effective for gathering information from the respondents. There were about 2308, 1530, and 1314 household farmers in Shina, Bebekis, and Kuhar Michael sub-districts, respectively. Among the 5152 household farmers in the sampled sub-districts, 2221, 1477, and 793 farmers were practicing irrigation agriculture in Shina, Bebekis, and Kuhar Michael sub-districts, respectively. Then the lists of the study population who are using irrigation agriculture were collected from the three sub-districts’ agriculture offices, and the sample size per sub-district was determined based on the number of farmers working on irrigation agriculture in each sub-district.

A simplified formula [29] was used to determine the total sample size from selected rural sub-districts, assuming a confidence level of 95 percent (with 0.05 margin of error).

                n = N / (1 + N e^2^)

Where n = the sample size

N = the population size, and

e = the level of precision/ tolerable sampling error.

Accordingly, a total of 380 representative farmers (368 based on the above formula and 12 additional samples for non-returned questionnaires for various reasons) were selected from the lists of the study population using a simple random sampling technique. The individual representative farmers were randomly selected with a lottery method.

The questionnaire were translated into the local language, Amharic, for easy communication. To check the level of clarity, wording, and understandability of the questions, a pilot test was made by inviting 18 farmers from Woreta zuria Sub-district of the District using convenience sampling methods. Thus, data from the respondent farmers were collected using pre-tested and structured questions having four sections: demographic information, knowledge, attitude, and practices questions about the effects of pesticide residues on humans and ecosystems.

The demographic characteristics included sex, age, and educational status of the respondents. Both the age and education levels of the respondents were grouped into five categories. All rural respondents were farmers working on irrigation agriculture and had more than a year of experience in pesticide application and use.

The KAPs of farmers about the effects of pesticide residues on ecosystems and humans were collected in a face-to-face mode by six trained extension workers of the Sub-districts with close supervision by three supervisors and the researcher.

A questionnaire consisting of seven items of knowledge and nine items of practices was presented to evaluate the knowledge and practices of the respondents, and the responses were coded as yes (scored 1) or no (scored zero). The attitudes of respondents were assessed using six Likert scale questions having five rating scales ranging from very low (one) to very high (five) scores [30–33].

Data collected from farmers using the close-ended questionnaire were complemented by open-ended questions and FGD from sampled farmers. This was to secure in-depth and reliable data on the KAPs of farmers about the effects of pesticide residues on ecosystems and humans. The numbers of FGD participants in Shina, Bebekis, and Kuhar Michael Sub-districts were 11, 11, and 7, respectively, and discussions were carried out in each Sub-district. In addition to the questionnaire and FGD, data were also collected from repeated field surveys supported with photos and videos.

Similarly, data about the negative effects of pesticide residues on ecosystems and humans consuming agricultural products were gathered from experts of Fogera District working in the agriculture office and from other sector offices, including Environmental Protection, Livestock, Cooperative, Water and Energy, and Land Administration and Use. The experts in these offices were selected purposively, assuming that they had sufficient experience with the effects of pesticide residues on ecosystems and humans in the area. Furthermore, they were consumers of agricultural products supplied by farmers. The names of the 151 experts in the offices were listed on a slip of paper and samples were selected using a simple random sampling technique following the same procedure to sample selection of the respondents in the rural Sub-districts. Based on a comparative study made among officers of Ethiopia and Hungary, 74%, 82%, and 77% of Ethiopian extension officers had knowledge, perception, and experience about pesticide toxicity, water, soil, and air pollution from pesticide residues and risks of pesticides to users, respectively [34].

From the pilot study using 15 invited experts, 86.67% knew about pesticides’ risks to ecosystems and humans. So the sample size of experts was determined, assuming that 86.67% of experts knew about the risks of pesticides to ecosystems and humans with 95% confidence level and 5% margin of error. The sample size was determined using the population proportion formula [34–36].


$$n=\frac{z^{2}x\;p\;x\;q\;x\;N}{e^{2}(N‐1)+z^{2}\;x\;p\;x\;q}$$

Where

**Table:** 

N =	Total number of experts in the sector offices
n =	Sample size
e =	Acceptable error(precision) which equals to 0.05
z =	The corresponding value to 95% confidence level which is equal to 1.96
p =	Proportion of officers with knowledge of pesticide applications(0.8667)
q =	Proportion of officers having no knowledge of pesticide application (0.1333)

Based on the formula, 82 respondent experts were selected using simple random sampling techniques. Similar to that of farmers, a questionnaire consisting of seven items of knowledge and nine items of practices was presented to evaluate the knowledge and practices of the experts, and the responses were coded as yes (scored 1) or no (scored zero). The attitude of respondents was assessed using six Likert scale questions having five rating scales ranging from very low (one) to very high (five) scores. In addition to close-ended questions, data was gathered from key informant experts using unstructured interview guides.

### 2.3. Data analysis

Data analyses were conducted using descriptive statistics, and the means, frequencies, and percentages were calculated to level the KAPs of the two groups of respondents. The response to open-ended questions was grouped and summarized based on similarities of responses. Binary and ordinal logistic regressions were used to measure the relationships between independent and dependent variables within the group. The odds ratios from the logistic regressions were used to level the strength of associations between demographic, knowledge, attitude, and practices related variables among respondents. The responses to items of KAPs’ questions were computed using SPSS version 26. The 95% confidence interval with an alpha-value less than 0.05 were accepted as statistical significance. The KAPs items for the two independent groups were transformed and tested using a two-sample independent t-test to check whether there was a significant KAPs difference between them regarding the effects of pesticide residues on humans and ecosystem health.

### 2.4. Ethical statement

This research work is part of dissertation research entitled Risks of Agricultural Pesticides to Aquatic Biodiversity and Consumers: A Case of Fogera District of Lake Tana Biosphere Reserve, Ethiopia. The main objective of the dissertation research work is to assess the knowledge, attitudes, and practices (KAPs) of farmers and consumers about the effects of pesticide residues and determine the level of risks of pesticide residues to aquatic biodiversity and human.

Hence, the objectives and the methodologies of the dissertation research work, including this study, were thoroughly evaluated by the council of graduate studies of the School of Fisheries and Wildlife of Bahir Dar University and approved. All the field works of the study were detailed in the methodology part of the research proposal, and the ethical board of Bahir Dar University reviewed and approved the implementation of the study. Prior to any data collection, information about the purpose of the study and the confidentiality of the data gathered from the respondents were communicated with the respondents, and they agreed to be interviewed. The respondents’ agreement is verbal and recorded as a witness of an agreement by the authors using a digital camera. Written data from the open and close-ended questionnaires were gathered using a structured questionnaire.

## 3. Results

### 3.1. Demographic information of respondents

The survey data were gathered from farmers of the three rural Sub-districts and experts of the six sector offices of Fogera District. The demographic information of the respondents is summarized below (Table 1).

**Table 1:** Demographic data of sampled farmers and experts.

R		Sex ratio			Age categories					Level of education				
		M	F	T	<25	28–35	36–45	46–55	<55	1	2	3	4	5
F	N	319	51	370	7	59	158	107	39	81	270	9	10	-
	%	86.2	13.8	100	1.9	15.9	42.7	28.9	10.5	21.9	73	2.4	2.7	-
E	N	67	15	82	2	39	32	8	1	0	0	0	8	74
	%	81.7	18.7	100	2.4	47.6	39	9.8	1.2	0	0	0	9.8	90.2

Note:—R = respondents, F = farmers, E = experts, 1 = illiterate, 2 = general primary, 3 = secondary school, 4 = diploma, 5 = first degree and above

A total of 370 farmers and 82 experts responded completely to the questionnaires. Among the 370 respondent farmers, 86.2% were males, and 42.7% were between the ages of 36–45 years. The education levels of most farmers (73%) were under the upper primary school level. Similarly, 81.7% of respondent officers of Fogera District were males, and most of them (47.6%) were in the age range of 26–35 years. Based on the years of work experience, 9.8%, 30.5%, and 59.8% of the experts had less than 5 years, 6 to 10 years, and above 10 years, respectively.

### *3*.2. Knowledge and attitudes of respondents

The results of the analysis showed that among the respondent farmers, 47%, 38.6% and 41.4% had knowledge about the effects of pesticide residues on aquatic life, the toxicity of pesticides they used, and the effects of fishing by pesticide poisoning on aquatic biodiversity, respectively. Similarly, 51.6%, 44.1%, 60.5% and 63.5% knew the effects of pesticide residues in vegetables and khat on consumers, the effects of pesticide residues in dead insects on birds feeding on the dead insects, the effects of pesticide residues in forage on animals and the transfer of pesticide residues across the food chains/webs, respectively. The results of the analysis of the knowledge of experts about the effects of pesticide residue on humans and ecosystems were also assessed using the same questions and are presented in Table 2.

**Table 2:** Knowledge of respondents about the effects of pesticide residues on human and ecosystem health.

Variables	Respondent farmers				Respondent Experts			
	N	R	F	%	N	R	F	%
Do you know about the negative effects of pesticide residues drained into the water, during the mixing of pesticides and washing sprayer equipment on aquatic life?	370	yes	174	47	82	Yes	66	80.5
		No	196	53		No	16	19.5
Do you know the level of toxicity of pesticides used by farmers for different purposes?	370	yes	143	38.6	82	Yes	65	79.3
		No	227	61.4		No	17	20.7
Do you know the negative effects of fishing by pesticide poisoning on aquatic living organisms?	370	yes	153	41.4	82	Yes	67	81.7
		No	217	58.6		No	15	18.3
Do you know about the negative effects of pesticide residues in the pesticide-sprayed vegetables and khat crops on consumers?	370	yes	191	51.6	82	Yes	70	85.4
		No	179	48.4		No	12	14.6
Do you know the negative effects of pesticide residues in insects killed by pesticides on birds consuming the dead insects?	370	yes	163	44.1	82	Yes	68	82.9
		No	207	55.9		No	14	17.1
Do you know the poisoning or killing effects of pesticide residues on animals when they feed on sprayed crops or crop residues?	370	yes	224	60.5	82	Yes	76	92.7
		No	146	39.5		No	6	7.3
							
Do you know about the transfer of pesticide residues across food chains/webs?	370	yes	235	63.5	82	Yes	71	86.6
		No	135	36.5		No	11	13.4

Note: N = total number of respondents, R = response, f = frequency

Source; field survey, 2022

Similarly, the attitude of the respondents towards the effects of pesticide residues on humans and ecosystems was analyzed and is presented in Table 3. Among the respondents, 47.8%, 47.8%, and 54.9% of farmers rated the desire of farmers to spray highly toxic pesticides on crops, the exposures of consumers to the effects of pesticide residues and the effects of pesticide residues drained into water bodies on aquatic life from high to very high, respectively. Furthermore, 32.7%, 27.6%, and 38.1% of farmers rated the effects of pesticide residues on soil-inhabiting living organisms, the interests of consumers to use vegetables harvested from the district, and the overall disruptive effects of pesticide residues on ecosystems from high to very high, respectively. The attitude of experts about the effects of pesticide residues on ecosystems and humans were also rated, and it is presented in the same table (Table 3).

**Table 3:** The attitude of respondents towards effects of pesticide residues on humans and ecosystem health.

Variables	R		Rating scales									
			1		2		3		4		5	
	G	N	N	%	N	%	N	%	N	%	N	%
How do you rate the desires of farmers to spray highly toxic pesticides on vegetable and khat crops to improve the glossiness of leaves?	F	370	51	13.8	61	16.5	81	21.9	120	32.4	57	15.4
	E	82	2	2.4	8	9.8	22	28.8	23	28	27	32.9
How do you rate exposures of consumers to the effects of pesticide residues from the consumption of pesticide-sprayed crops supplied by farmers?	F	370	29	7.8	52	14.1	112	30.3	96	25.9	81	21.9
	E	82	0	0	3	3.7	15	18.3	27	`32.9	37	45.1
How do you rate the negative effects of pesticide residues drained into water bodies during mixing it and washing the sprayer equipments on aquatic life?	F	370	32	8.6	55	14.9	80	21.6	136	36.8	67	18.1
	E	82	1	1.2	6	7.3	10	12.2	19	23.3	46	56.1
How do you rate the negative effects of pesticide residues on soil-inhibiting organisms?	F	370	20	5.4	97	28.2	132	35.7	91	24.6	30	8.1
	E	82	0	0	12	14.6	20	24.4	28	31.7	24	29.3
How do you rate your overall interest to use vegetable products harvested from the study area?	F	370	39	10.5	97	28.2	132	35.7	75	20.3	27	7.3
	E	82	0	0	20	24.4	24	29.3	19	23.2	18	22
How do you rate the overall ecosystems disrupting effects of pesticide residues in the study area?	F	370	18	4.9	95	25.7	116	31.4	97	28.2	44	11.9
	E	82	1	1.2	20	24.4	24	29.3	19	23.3	18	22

Note: R = respondents, 1 **=** very low, 2 = low, 3 = Average, 4 = high, 5 = very high, G = group, F = farmers, N = Total sample, E = experts, n = number of respondents per level of scale

Source; field survey, 2022

### 3.3. Observations and practices of respondents

The observations and practices of respondents were summarized and presented in Table 4. Among the total respondents, 84.6%, 77.3%, 44.9%, and 70% of farmers responded that they mixed pesticides near water bodies, washed pesticide sprayer equipment near water bodies, sprayed highly toxic pesticides on crops, and used prohibited pesticides to store crops, respectively. Furthermore, 61.6%, 76.8%, 69.2%, 59.5%, and 61.9% of farmers responded that farmers supplied vegetables without waiting for the required time interval between pesticide spraying and crop supplying, used vegetables without waiting for the required time interval after spraying, used the unsold vegetables for their own home consumption, used unsold vegetables for their livestock feed and consumed vegetables without any health concern, respectively. Similarly, the observations and practices of experts were analyzed and is presented.

**Table 4:** Responses of target groups to items of observation and practices.

Variables	Respondent farmers				Respondent Experts			
	N	R	F	%	N	R	F	%
Do farmers mix their agricultural pesticides near water bodies	370	Yes	313	84.6	82	yes	70	85.4
		No	57	15.4		No	12	14.6
Do farmers wash pesticide spraying equipment near water bodies	370	Yes	286	77.3	82	yes	71	86.6
		No	84	22.7		No	11	13.4
Do farmers use spraying highly toxic chemical pesticides to control crop pests and shine the leaves of some crops?	370	Yes	166	44.9	82	yes	54	65.9
		No	204	55.1		No	28	34.1
Do farmers apply prohibited pesticides to store crops for longer period of time?	370	Yes	259	70	82	yes	60	73.2
		No	111	30		No	22	26.8
Do farmers supply vegetables and khat without waiting for the required time interval between the pesticide spraying and the crops supplying?	370	Yes	228	61.6	82	yes	57	69.5
		No	142	38.4		No	25	30.5
Do farmers use vegetables without waiting for required time interval between pesticide spraying & crops supplying?	370	Yes	284	76.8	82	yes	45	54.9
		No	86	23.2		No	37	45.1
							
Do farmers use the unsold vegetables for their own home consumption?	370	Yes	256	69.2	82	yes	46	56.1
		No	114	30.8		No	36	43.9
Do farmers feed the unsold vegetables for their livestock?	370	Yes	220	59.5	82	yes	34	41.5
		No	150	40.5		No	48	58.5
Do you use all types of vegetables without concern about health risks from pesticide residues?	370	Yes	229	61.9	82	yes	25	30.5
		No	141	38.1		No	57	69.5

Note: N = Total number of respondents, R = response, f = frequency

Source; field survey, 2022

### 3.4. Relationship between demographic factors and KAPs of farmers

The relationships between demographic factors and farmers’ knowledge about the effects of pesticides on ecosystems and humans were analyzed using binary logistic regression and are presented in Table 5. The younger farmers (age groups < = 35) were less likely to know about the negative effects of pesticide residues in water bodies on aquatic life, on food crops on consumers, and in forage on animals (OR = 0.325; 0.356 & 0.171). Females were more likely to know about the negative effects of pesticide residues in food crops on consumers and the negative effects of fishing by pesticide poisoning than males (OR = 2.23 & 2.301). The ages of farmers ranging from 46 to 55 were more likely to understand the transfer of pesticide residues across food chains than farmers with the rest age groups (OR = 2.196).

**Table 5:** Results showing the relationship between demographic factors and knowledge of farmers.

Explanatory(Demographic) variables	Outcome variables (Knowledge about):	B	S.E.	Wald	Df	Sig.	Exp (B)	95% C.I.for EXP(B)	
								Lower	Upper
Respondents Age (< = 35)	Negative effects of pesticides residues that drain into water bodies on aquatic life	-1.125	.419	7.199	1	.007	.325	.143	.738
Respondents Sex (female)	Level of toxicity of pesticides used for different purposes	-.707	.351	4.066	1	.044	2.028	1.020	4.032
respondents Sex (female)	Negative effects of fishing by pesticide poisoning on aquatic organisms	.833	.345	5.847	1	.016	2.301	1.171	4.522
Respondents Sex(female)	Negative effects of pesticide residues in vegetable and khat on consumers	.802	.320	6.294	1	.012	2.230	1.192	4.174
Respondents Age(< = 35)		-1.032	.421	6.012	1	.014	.356	.156	.813
Respondents Age (< = 35)	Negative effects of pesticide residues in crop/ residues on animals feeding it	-1.764	.461	14.653	1	.000	.171	.069	.423
Respondents Age (36–45)		-.754	.362	4.343	1	.037	.471	.232	.956
Respondents Age (46–55)	Transfer of pesticide residues across the food chain	.787	.391	4.049	1	.044	2.196	1.021	4.725

**Note:** As respondents in the lower age category (age groups < 25) are very small (7 only), it is merged into the next age category for analysis, and the last age category and males were used as a reference

The relationship between the respondent farmers’ knowledge and attitudes about pesticide residues’ effects was also analyzed and is presented in Table 6. Farmers having knowledge about the negative effects of pesticide residues in water bodies on aquatic life, in foodstuff on consumers, and in animal forage on livestock had more desire to spray vegetables with toxic pesticides (OR = 2.02; 2.904 & 2.272). Farmers having knowledge about the negative effects of pesticide residues in foodstuffs on consumers and in forage on livestock and the transfer of pesticide residues across the food chain had higher beliefs and feelings on exposure of consumers to effects of pesticide residues than farmers having lower knowledge (OR = 3.539, 2.374 & 2.919). Similarly, farmers having knowledge about the negative effects of pesticide residues on consumers and livestock had higher beliefs and feelings about the negative effects of pesticide residues in water bodies on aquatic life than respondents with lower knowledge (OR = 2.542 & 2.144). Farmers that knew about the level of toxicity of pesticides had higher beliefs and feelings about the negative effects of pesticide residues on soil-inhibiting organisms than farmers having lower knowledge about the level of toxicity (OR = 2.004).

**Table 6:** Relationships between knowledge and attitudes of respondents about effects of pesticide residues.

Explanatory Variables Knowledge about:	Outcome variable (Attitudes)	B	Std. Error	95% Wald C.I.		Hypothesis Test			Exp (B)	95% Wald C.I. for Exp(B)	
				Lower	Upper	Wald χ^2^	df	Sig.		Lower	Upper
Negative effects of pesticide residues that drain into water bodies at time of mixing and equipment’s’ washing on aquatic life	Desires of farmers to spray toxic pesticides on vegetable and khat crops to improve their glossiness	.703	.3009	.113	1.293	5.460	1	.019	2.020	1.120	3.644
Negative effects of pesticide residues in vegetable and khat crops on consumers		1.066	.2768	.524	1.609	14.838	1	.000	2.904	1.688	4.996
Negative effects of pesticide residues in crops /crop residues on animals feeding		.821	.2780	.276	1.365	8.711	1	.003	2.272	1.317	3.917
Negative effects of pesticide residues in vegetable and khat crops on consumers	Consumers exposure to effects of pesticide residues from pesticide sprayed crops supplied by farmers of the study area	1.284	.2818	.712	1.816	20.114	1	.000	3.539	2.037	6.149
Negative effects of pesticide residues in crops /crop residues on animals feeding it		.865	.2808	.314	1.415	9.483	1	.002	2.374	1.369	4.117
Transfer of pesticide residues across food chain		1.071	.2802	.561	1.581	16.948	1	.000	2.919	1.753	4.860
Negative effects of pesticide residues in vegetable and khat crops on the consumers?	Negative effects of pesticide residues drain into in water bodies on aquatic life	.933	.2882	.407	1.459	12.106	1	.001	2.542	1.503	4.300
Negative effects of pesticide residues in crops / crop residues on animals feeding		.763	.2899	.234	1.292	7.985	1	.005	2.144	1.283	3.639
Level of toxicity of pesticides used by farmers for different purposes?	Negative effects of pesticide residues on soil inhibiting organisms	.695	.3405	.028	1.362	4.167	1	.041	2.004	1.028	3.906

Similarly, the result of the analysis of relationships between the knowledge and practices of respondent farmers about the effects of pesticide residues was summarized and presented in Table 7. Farmers having knowledge about the effects of pesticide residues in vegetables and khat crops on consumers were less likely to mix pesticides near water bodies (OR = 0.410). Farmers having knowledge about the effects of pesticide residues on aquatic life that drained from pesticide mixing and sprayer equipment washing near water bodies were less likely to wash pesticide sprayer equipment near water bodies (OR = 0.513). Farmers having prior knowledge about the transfer of pesticide residues across the food chain were less likely to apply prohibited pesticides on stored crops to preserve it for longer periods of time (OR = 0.455). Farmers having knowledge about the toxicity of pesticides used for various purposes were less likely to supply vegetables without waiting for the required time interval between spraying and supplying (OR = 0.553). Farmers having knowledge about the negative effects of pesticide residues contained in forage on livestock were less likely to supply vegetables without waiting for the required time interval between spraying and supplying, to use vegetables without waiting for the required time interval between pesticide spraying and crops supplying, to use unsold vegetables for their home consumption and their livestock feed and to use all types of vegetables without concern about health risks from pesticide residues (OR = 0.535, 0. 368, 0.361, 0.388 & 0.377).

**Table 7:** Relationships between the knowledge and practices of respondents about the effects of pesticide residues.

Explanatory variables Knowledge about:	Outcome variables Farmers have been:	B	S.E.	Wald	Df	Sig.	Exp (B)	95% C.I.for EXP(B)	
								Lower	Upper
Negative effects of pesticide residues in vegetable and khat crops on consumers(yes)	mixing pesticides near water bodies	-.892	.303	8.660	1	.003	.410	.228	.742
Negative effects of pesticides residues on aquatic life that drained into water bodies during pesticide mixing & equipment washing (yes)	washing pesticide sprayer equipments near water bodies	-.668	.258	6.695	1	.010	.513	.309	.850
Transfer of pesticide residues across the food chain/ web (yes)	applied prohibited pesticides on stored crop to protect longer period	-.787	.233	11.453	1	.001	.455	.288	.718
Level of toxicity of pesticides used by farmers for various purposes (yes)	supplying vegetables without waiting for the required time interval between spraying and supplying	-.592	.237	6.251	1	.012	.553	.348	.880
Negative effects of pesticide residues in crops or crop residues on animals feeding it (yes)		-.625	.227	7.606	1	.006	.535	.343	.835
Negative effects of pesticide residues in crops or crop residues on animals feeding it (yes)	using vegetables without waiting for the time interval between spraying & supplying	-1.001	.252	15.763	1	.000	.368	.224	.603
Negative effects of pesticide residues in crops or crop residues on animals feeding it (yes)	Using unsold vegetables for own home consumption	-1.018	.392	6.754	1	.009	.361	.168	.779
Negative effects of pesticide residues in crops or crop residues on animals feeding it (yes)	using unsold vegetables for their livestock feed	-.981	.220	19.864	1	.000	.375	.244	.577
Negative effects of pesticide residues in crops or crop residues on animals feeding it (yes)	Using all types of vegetables without concern about health risk from pesticides residues	-.977	.222	19.424	1	.000	.377	.244	.581

**Note:** zero/Not is used as a reference category

The result of the analysis of the independent t-test about KAPs of respondent farmers and experts is presented in Table 8. The test result confirmed that there was a significant knowledge difference between the two independent groups (t (450) = -10.051, p = 0.000) about the effects of pesticide residues on ecosystems and consumers. The mean value of experts (M = 0.8415, 1 = yes, SD = .26561) was higher than the mean value of farmers (M = 0.4954, 0 = not, SD = 0.34692), and the magnitude of the difference in mean (mean difference = -0.34610, 95% CI: 0.27806 to 0.41414) was significant. Like that of knowledge, there was a significant difference in attitudes of farmers and experts towards the effects of pesticide residues on ecosystems and humans (t (450) = -6.529, p = 0.000). The mean value of experts (M = 3.8069, 4 = high, SD = 0.81879) was higher than the mean value of farmers (M = 3.1766, 3 = average, SD = 0.78475), and the magnitude of the difference in mean (mean difference = -0.63033, 95% CI: -.82007 to -.44060) was significant. Similarly, there was a significant difference in practices and observations of respondents regarding mixing the pesticide near water bodies, washing the sprayer equipment, using highly toxic and prohibited pesticides, and the pesticide-spraying and crops-supplying practices of farmers without waiting for the post-spray time interval (t (450) = -2.333, p = 0.020). The mean value of experts (M = .7610, 1 = yes, SD = .29387) was higher than the mean value of farmers (M = .6768, SD = 0.29623), and the magnitude of the difference in mean values (mean difference = -0.08422, 95% CI: -.15517 to -.01326) was significant. Similarly, there was a significant difference in practices and observation of the two independent groups on using pesticide-sprayed agricultural products and crop residues for the producers themselves and their livestock feed (t (450) = 4.352, p = 0.000). Here the mean value of farmers (M = .6682, 1 = yes, SD = .39506) was higher than experts (M = .4573, 0 = not, SD = 0.40598), and the magnitude of the difference in mean (mean difference = .21093, 95% CI: .11569 to .30617) was significant.

**Table 8:** Difference in KAPs of respondent groups using independent samples t-test.

Variables		Levene’s Test for Equality of Variances					t-test for Equality of Means										
		Mean	SD	F	Sig.	T		Df		Sig. (2-tailed)		Mean Diff.	Std. Error Diff.		95% CI of the Diff.		
															Lower		Upper
Knowledge	G1	.4954	.34692	18.738	.000	-8.496			450	.000	-.34610			.04074		-.42615	-.26604
	G2	.8415	.26561			-10.051			149.146	.000	-.34610			.03443		-.41414	-.27806
Attitude	G1	3.1766	.78475	.168	.682	-6.529			450	.000	-.63033			.09654		-.82007	-.44060
	G2	3.8069	.81879			-6.354			116.278	.000	-.63033			.09920		-.82680	-.43386
observation/ practices	G1	.6768	.29623	.929	.336	-2.333			450	.020	-.08422			.03610		-.15517	-.01326
	G2	.7610	.29387			-2.345			120.250	.021	-.08422			.03592		-.15534	-.01310
observation/ practices	G1	.6682	.39506	.013	.908	4.352			450	.000	.21093			.04846		.11569	.30617
	G2	.4573	.40598			4.277			117.428	.000	.21093			.04931		.11327	.30859

Note:- 1 = yes, 0 = no for knowledge and observation/ practices, and a 5 level Likert scale for attitude ranging from 5(very low) to 1(very high), G1 = farmers, G2 = experts.

### 3.5. Field observations and focused group discussions (FGD)

The farmers called the pesticide as “Medihanit” in their local language, which means medicine, and some of them believed that these pesticides targeted only the pests. Though most farmers attended the awareness creation training given by different stakeholders and knew about the health risks of pesticides, they didn’t take any safety measures to reduce the negative effects of pesticide residues on consumers and ecosystems. Since most farmers didn’t observe acute health effects from pesticides immediately after application practices, they didn’t believe what experts said about the long-term effects of pesticide residues. Even though farmers were sick due to acute poisoning after applications, they didn’t correlate the causes of illness with pesticide residues. But all the FGD participant farmers agreed on the ever-increasing occurrence of diseases, including blood cancer, blindness, and diabetes among farmers in the study area, which were not common in the past. The FGD participants also mentioned that the community in the study area became more sensitive and loss its tolerance to any irritants. The open access of pesticides to everybody without recommendation and any regulatory practices exposed communities, particularly women and children, to self-poisoning and suicide at times of minor conflict within the family. Consequently, the health officials of the District confirmed that pesticide was one of the top human health risks.

The observation of experts also confirmed that most farmers had sprayed pesticides without wearing any safety equipment and taking any care to protect the ecosystems. Moreover, it was noted that most farmers discarded the empty pesticide containers into the field, including water bodies, and released the spray leftover pesticides on the soil or into water bodies.

There were some good practices of collecting selected empty pesticide containers and using them for recycling purposes. Some individuals in the study area have collected and crushed the empty pesticide containers to sell them to plastic recycling projects. Nevertheless, the recycling practices of empty pesticide containers were not properly designed to manage the effluents not to pollute the groundwater in the study area. The containers of highly persistent pesticides were also used for recycling.

Most farmers knew about the transfer of pesticide residues through food sources from animals to humans and didn’t use the milk and/or meat of animals if the animals were suspected of feeding on plants sprayed by pesticides. However, the majority of the farmers didn’t know about the effects of pesticide residues on other ecosystem components. When they were asked about the extent of the effects of pesticide residues on birds that fed on insects killed by pesticide poisoning, they defended against birds of seed eaters that were in conflict with farmers.

It was confirmed that some farmers used highly toxic pesticides such as diazinon, chloropyrifos, malathion 50% EC, Nativo, and profenofos deliberately to improve the freshness of vegetables and soften the leaves of chewing khat without worrying about their negative effects on consumers. They sprayed the pesticides on vegetables and supplied them without waiting for the time interval after spraying. Most consumers of Woreta town knew the practices of producers and had stopped using cabbage, Ethiopian cabbage, green pepper, and tomato. As a result, the major users of vegetables in the study area were residents of Bahir Dar and Debretabor cities which are 55km and 40km away from Woreta town.

There were acute poisoning and death of animals such as rats, birds, cats, goats, cattle, hyenas and fish in the study area. The possible reasons for poisoning and death were treatment of exoparasites of animals, induced animal food poisoning and transfer of pesticide residues across the food chain/webs. The fishermen chopped vegetables, socked them into pesticides such as malathion, and fed on the fish by dropping the chopped vegetables into ponded river water during dry seasons. The fish in different age groups were killed by the poisoning, and then birds fed on the dead fish. This induced poisoning of tributaries and rivers was one of the possible factors for the fish production decline and might cause aquatic species extinction if the practices continue. The fish was harvested immediately after death and supplied to consumers within a short time after harvest.

In addition to the deliberate poisoning of aquatic life, a large amount of pesticide residues was drained into tributaries during the mixing of pesticides and washing of the sprayer equipment at the edge of tributaries and major rivers. The pesticide residues might severely impair the aquatic organisms and their habitat elements. There were also symptoms of risks of surface water pollution, which was used for drinking in the study area. Unlike the past 20 to 30 years, drinking surface and pond water in most irrigated localities of the study area caused vomiting and diarrhea immediately after drinking. It was assumed that the reason for vomiting and diarrhea after drinking surface water in the study area was water pollution from pesticide residues. There were also human deaths from eating pesticide-sprayed raw vegetables such as tomatoes in the field and from the application of toxic pesticides on stored crops. A farmer in the study area lost his three children at a time by feeding them crops contaminated by rodenticides to control pests in his store. Farmers have applied similar types of pesticides on crops in store and supplied it to consumers without waiting for the time required to lose their toxicity. The absence of a responsible body to control and regulate the pesticide supply chain at the District level and failarity of the pesticide task force to accomplish their responsibilities elevated the risks of pesticide residues.

## 4. Discussion and implications

More than 64% of the farmers didn’t know about the level of toxicity of pesticides they used. Similar studies conducted in different parts of Ethiopia also confirmed that lack of knowledge about the safe use of pesticides was the major reason for using banned and highly toxic pesticides on food crops [14, 37, 38]. Similarly, more than half of the farmers didn’t know about the effects of pesticide residues on aquatic life and on birds feeding on insects killed by the pesticide poisons. On the contrary, they had better knowledge about the effects of pesticide residues on livestock feeding on forage contaminated with pesticide residues and the transfer of pesticide residues across the food chain. The knowledge of farmers about the effects of pesticide residues was influenced by some demographic factors such as age and gender. The elderly farmers had better knowledge about the negative effects of pesticide residues than the younger ones. They knew about the transfer of pesticide residues across the trophic level more than younger groups which might be due to life experiences and pieces of training given by NGOs and agricultural extension workers. The agriculture office, MEDA project and AGROBIG project have given awareness-raising training to landholders, and fortunately, most of the landholder farmers were the elder ones.

Regarding gender, female farmers had better knowledge about the negative effects of pesticide residues on consumers than males. The reason for such a better understanding of females about the negative effects of pesticide residues on consumers might be their exposure to challenges of consumers in the market center and higher chance to observe the effects of contaminated food on the family when they used it without waiting for the required time interval between pesticide application and crop harvest. Females were consumers’ major suppliers of vegetable products.

Though 47% and 54.9% of farmers had knowledge and high belief on the effects of pesticide residues on aquatic life, more than 77% of them mixed the pesticides and washed their sprayer equipment near water bodies. Similar studies conducted in different parts of the country also confirmed that more than 70% of sampled farmers mixed their pesticides near water bodies [23, 39, 40]. The knowledge gaps about the effects of pesticide residues on humans and ecosystems were not the only factor in mixing pesticides and wash the sprayer equipment near water bodies. The numbers of farmers that mix pesticides and wash sprayer equipment near water bodies were greater than the number of farmers having knowledge gaps about the negative effects of pesticide residues. Though most farmers had knowledge about the negative effects of pesticide residues on the environment and non-target species, they didn’t undertake any precautions in the application processes [41]. Regardless of the knowledge of farmers about the negative effects of pesticide residues, they had a strong desire to use highly toxic chemicals to store crops and improve the glossiness of vegetables and khat. The desire to use highly toxic pesticides was common to all farmers in the study area. However, the desire to use it was much more among farmers having knowledge about the negative effects of pesticide residues. Previously conducted studies confirmed that farmers had used highly toxic pesticides, including organochlorine pesticides, to control pests and improve the qualities of Khat leaves and vegetables [23, 38, 42]. According to [43], more than 58% of pesticides registered in Ethiopia, including in the study area, were under the list of highly hazardous pesticides. Such pesticide residues in vegetables might pose serious human health problems [44]. Unless pest resistance crop varieties are introduced or pesticides with minimal negative effects are supplied, farmers would use highly toxic pesticides, and their effects would be severer than ever before.

Though more than half of the farmers (51.6%) knew about the effects of pesticide residues in vegetables and khat on consumers, less than 40% of them supplied the crops by waiting for the required time interval between pesticide spraying and crops supplying. It implies that the farmers who knew about pesticide residues’ negative effects on consumers were supplying crops without waiting for the time required between spraying and supplying. The results of the logistic regression also confirmed that knowledge had no significant impact on the reduction of farmers’ desire to use highly toxic pesticides. Thus, only awareness raising is not enough to bring the intended behavioral change among farmers. Awareness raising should be coupled with effective law enforcement to minimize the effects of pesticide residues on humans and ecosystems. Furthermore, there should be a responsible body/organization at District and Sub-district levels to regulate the pesticide supply chain and its applications [9]. Unless pesticide-supplying, spraying and crop-supplying systems are regulated effectively, the strong desire of farmers to use highly toxic pesticides will worsen the negative effects of pesticide residues on ecosystems and consumers.

Among the respondents, 47.8% of farmers and 78% of experts believed that pesticide residues in food crops might cause health risks to consumers. More than 69% of the vegetable consumers in the study area had a concern about the health risks from pesticide residues. Previous studies also indicated that contamination of potable water and food crops from pesticide residues are becoming a major health concern for consumers globally [9, 45, 46]. The study confirmed that there were symptoms of surface water pollution from pesticide application practices of farmers in the study area to the extent that cause vomiting and diarrhea to water users immediately after drinking [47]. A study conducted by [48] in Robit Bata of the Lake Tana Watershed confirmed that there was surface and groundwater pollution by persistent pesticides such as chlorpyrifos and endosulfan. A study conducted in the Central Rift Valley of Ethiopia using GC-MS indicated that there was at least one or more pesticide residues per food item, and the amount of pesticide residues in one-third of the sampled food items were above the maximum residual limits [49]. The intoxicating pesticides used by horticulture farmers and persistent pesticides used in stores of crops were major sources of food poisoning [50]. The consumers of Woreta town who knew about the effects of pesticide residues and the pesticide spraying and crop-supplying practices of farmers had either ceased or reduced vegetable consumption. This is an important indicator for the need to conduct pesticide risk monitoring to ensure food safety [51, 52]. If the trend of pesticide spraying and crop-supplying practices continues, it will have serious health risks to consumers and will have negative implications on the vegetable supply chain in the near future.

Though the effects of pesticide residues are highly life-threatening [7, 8, 11, 14, 17, 53, 54], less than half of the respondent farmers (38.1%) and experts (45.3%) had high to very high belief about the disrupting effects of pesticide residues on ecosystems. This study indicated that even experts had a limited understanding of the effects of pesticides on the ecosystems. Most members of the two groups of respondents believed that the pests of crops would be exterminated from the study area if the effects of the pesticide residues on soil-inhibiting organisms were severe. Previous studies confirmed that the effects of pesticide residues were life-threatening, and the severity of the effects of pesticide residues is dependent on types of species, age groups, sex, stressors and conditions of animals [54, 55]. In addition to acute effects of pesticide residues, they might damage the feed of larvae of aquatic organisms and decline their survival, disrupt their endocrine system, change the sex ratios and sexual characteristics of organisms and cause the failure of reproduction, inhibit growth and development of organisms and reduce the avoidance of organisms from their natural predators [7, 56–58]. In addition to effects from the physico-chemical parameters[59, 60], the pesticide residues might be one of the major factors for fish sex ratio difference and fish production decline in the study area. So the recurrent occurrence of pests of crops in the study area might be due to failure to use the recommended types and doses of pesticides or due to the development of pesticide-resistant pests [61]. Though Ethiopia declared industrial chemicals and agricultural pesticide registry and supply chain proclamations [62, 63], it failed to enforce the laws [17].

Most farmers responded that they consumed the vegetables sprayed with pesticides without waiting for the required time interval after spray. In contrast to farmers, more than 40% of experts believed and observed that farmers have been supplying crops with highly toxic pesticides to consumers but they did not use such sprayed crops for themselves and even for their livestock. Similar to this finding, the rice-producing farmers in China overused pesticides though they had knowledge about the toxicities of pesticides and its effects on the environment [64]. Farmers treated the crops for their own consumption and for sale separately. Most farmers (63.8%) knew about the transfer of pesticide residues through food to consumers and they abstained from using animal products (milk) during periods of intensive pesticide application to avoid themselves from pesticide poisoning, but they milked their cows and supplied the raw milk immediately or the butter to consumers of Woreta town. Consumption of such contaminated animals and their byproducts are the primary source of health risks to humans [13]. The ever-increasing prevalence of different diseases such as blindness, cancer, diabetes and hypersensitivities in the study area might be due to unsafe application practices of pesticides and pesticide residues in foodstuffs. Studies confirmed that pesticide residues might cause cancer, hormonal disruptions, developmental disorders, early maturity, sterility, loss of memory and coordination, reduced visual ability and motor skills, respiratory illness, endometriosis, autism, hypersensitivity, diabetes and obesity to humans [2, 58].

Most farmers knew about the effects and transfer of pesticide residues across the food chain, but their understandings of the effects of pesticide residues beyond themselves and their livestock were limited. Similarly, the knowledge gaps of farmers about the effects of pesticides were confirmed by different researchers [37, 38]. Most of the farmers didn’t know about the negative effects of pesticide residues on birds of prey and did not know about the feeding habits of different bird species. On the other hand, most experts knew about the death of insectivorous birds feeding on insects killed by pesticide poisoning and the effects of pesticide residues across the food chain.

The results of the odds ratio confirmed that the attitude of most farmers about the negative effects of pesticide residues on humans and ecosystems had a strong relationship with their prior knowledge. However, knowledge had no significant influence on changing the desire of farmers to use highly toxic and banned pesticides to control pests and improve the glossiness of vegetables and khat. Similarly, the practices of farmers had strongly influenced by their prior knowledge about the effects of pesticide residues to human and the environment. Most of the farmers that knew about the negative effects of pesticides residues on consumers supplied their crops waiting for the required time interval between spraying and supplying. However, half of the farmers had no knowledge about the effects of pesticide residues on ecosystems and humans, and some of the farmers having prior knowledge had deliberately sprayed highly toxic pesticides on their crops and supplied it without waiting for the required time interval.

The analysis of the independent t-test confirmed that there were significant KAPs differences between the two independent groups about the effects of pesticide residues on ecosystems and consumers. The knowledge and attitudes of experts about the effects of pesticide residues on ecosystems and humans were higher than that of farmers. Furthermore, the observations of experts confirmed that most farmers mixed the pesticides and washed sprayer equipment near water bodies, used highly toxic and prohibited pesticides and supplied crops without waiting for the post-spray time interval. Similarly, there was a significant difference in responses of the two groups with regard to using pesticide-sprayed agricultural products and crop residues for producers themselves and their livestock feed, respectively. The observation of experts confirmed that most farmers didn’t use the sprayed crops and crop residues for themselves and their animals, respectively. Awareness raising, experience, and expert recommendation services were important factors in knowing the negative effects of pesticide residues on ecosystems, and the practices of pesticide management were highly influenced by both knowledge and attitude [32, 65]. The FGD among experts confirmed that most producers didn’t use the pesticide-sprayed vegetables for themselves without waiting for the time interval between spraying and supplying. So the health risks of consumers from eating foodstuffs sprayed by toxic pesticides were higher than that of producers. In general, the effects of pesticide residues on humans and ecosystems were resulted from knowledge gaps among pesticide users and using the highly toxic pesticides purposively without worrying about the negative effects of pesticide residues. Level of education of the pesticide users, their knowledge about the effects of pesticides, regular monitoring schemes on pesticide supply chain and food safety, institutional capacity building and enforcement of laws are important measures to minimize the extent of risks of pesticides on humans and the environment [24, 38, 66–68].

## 5. Conclusions and recommendations

This study reveals that knowledge gaps on the negative effects of pesticides, deliberate use of highly toxic pesticides, pesticide spraying and crop supplying without waiting for the post-spray time interval, and drifting of pesticide residues to water bodies were the major factors that exposed humans and ecosystems to the effects of pesticide residues. There was also a strong desire of farmers to spray toxic chemicals on crops to protect it from pest infestation during storage and fishing practices from rivers by intoxicating the fish with a pesticide-contaminated feed. This practice might cause adverse effects on aquatic ecosystems and consumers. Most pesticides used in the study area were highly toxic to aquatic life, including fish. Thus, this might be one of the major factors for the fish sex ratio difference and the declining of fish production. In addition to its effects on ecosystems, pesticide residues on foodstuffs and surface water might expose consumers to serious health risks. Though they didn’t consume it for themselves, some farmers have been selling vegetables and chewing khat leaves that were harvested after being sprayed with pesticides not so long ago. This practice was done intentionally to prolong the shelf life of the vegetables and soften the leaves of khat without worrying about their negative effects on the consumer. Most consumers of Woreta town knew the practices of farmers and were concerned about their health risks from pesticide residues.

To minimize the negative effects of pesticides on humans and ecosystems, awareness-raising practices on their negative effects and management, a clear pesticide supply chain, prohibition of open access of pesticides, enforcement of laws, delineation of pesticide-free buffer zones for water bodies, and regular food safety monitoring mechanisms are highly required.

## Supporting information

S1 File (PDF)
