# Supplementary material for: Knowledge, attitude and practices of farmers and experts about the effects of pesticide residues on agricultural product users and ecosystems: A case of Fogera District, Ethiopia
Source: PLoS One. 2023 Dec 7;18(12):e0292838. doi: 10.1371/journal.pone.0292838 (PMC10703333; doi:10.1371/journal.pone.0292838)
Supplement: S1 File — (PDF) [file pone.0292838.s001.pdf]

Knowledge, Attitude and Practices of Farmers and Experts about the Effects of Pesticide Residues on Agricultural Product Users and Ecosystems: A case of Fogera District, Ethiopia

Abebaw Abaineh*, Dessalegn Ejigu, Minaleshewa Atlabachew, Eshete Dejen, and Gashaw Tilahun

November 2023

DATA FROM RESPONDENT FARMERS

Frequency Table of farmers' Demography

Statistics

		sex of respondents	age of respondents	level of education
N	Valid	370	370	370
	Missing	0	0	0

sex of respondents

		Frequency	Percent	Valid Percent	Cumulative Percent
Valid	male	319	86.2	86.2	86.2
	female	51	13.8	13.8	100.0
	Total	370	100.0	100.0	

age of respondents

		Frequency	Percent	Valid Percent	Cumulative Percent
Valid	<=25	7	1.9	1.9	1.9
	26-35	217	58.6	58.6	60.5
	46-55	107	28.9	28.9	89.5
	>55	39	10.5	10.5	100.0
	Total	370	100.0	100.0	

level of education

		Frequency	Percent	Valid Percent	Cumulative Percent
Valid	illiterate	81	21.9	21.9	21.9
	general primary	270	73.0	73.0	94.9
	secondary school	9	2.4	2.4	97.3
	diploma	10	2.7	2.7	100.0
	Total	370	100.0	100.0	

Frequency table of farmers' Knowledge

Statistics

		Do you know about the negative effects of pesticides residues, entering in to water bodies at time of mixing and washing equipment, to aquatic life?	Do you know the level of toxicity of pesticides used by farmers for different purposes?	Do you know the negative effects of fishing practices by pesticide poisoning to aquatic organisms including fish?	Do you know about the negative effects of pesticide residues in pesticide sprayed vegetable and khat crops to the consumers?	Do you know the negative effects of pesticide residues in insects killed by pesticides to birds eating them?	Do you know the negative effects of pesticide residues to animals feeding on pesticide sprayed crops and crop byproducts	Do you know about transfer of pesticide residues across the food chain?
N	Valid	370	370	370	370	370	370	370
	Missing	0	0	0	0	0	0	0
Minimum		0	0	0	0	0	0	0
Maximum		1	1	1	1	1	1	1

Do you know about the negative effects of pesticides residues, entering in to water bodies at time of mixing and washing equipment, to aquatic life?

		Frequency	Percent	Valid Percent	Cumulative Percent
Valid	not	196	53.0	53.0	53.0
	yes	174	47.0	47.0	100.0
	Total	370	100.0	100.0	

Do you know the level of toxicity of pesticides used by farmers for different purposes?

		Frequency	Percent	Valid Percent	Cumulative Percent
Valid	not	227	61.4	61.4	61.4
	yes	143	38.6	38.6	100.0
	Total	370	100.0	100.0	

Do you know the negative effects of fishing practices by pesticide poisoning to aquatic organisms including fish?

		Frequency	Percent	Valid Percent	Cumulative Percent
Valid	not	217	58.6	58.6	58.6
	yes	153	41.4	41.4	100.0
	Total	370	100.0	100.0	

Do you know about the negative effects of pesticide residues in pesticide sprayed vegetable and khat crops to the consumers?

		Frequency	Percent	Valid Percent	Cumulative Percent
Valid	not	179	48.4	48.4	48.4
	yes	191	51.6	51.6	100.0

	Total	370	100.0	100.0	

Do you know the negative effects of pesticide residues in insects killed by pesticides to birds eating them?

		Frequency	Percent	Valid Percent	Cumulative Percent
Valid	not	207	55.9	55.9	55.9
	yes	163	44.1	44.1	100.0
	Total	370	100.0	100.0	

Do you know the negative effects of pesticide residues to animals feeding on pesticide sprayed crops and crop byproducts

		Frequency	Percent	Valid Percent	Cumulative Percent
Valid	not	146	39.5	39.5	39.5
	yes	224	60.5	60.5	100.0
	Total	370	100.0	100.0	

Do you know about transfer of pesticide residues across the food chain?

		Frequency	Percent	Valid Percent	Cumulative Percent
Valid	not	135	36.5	36.5	36.5
	yes	235	63.5	63.5	100.0
	Total	370	100.0	100.0	

Frequency table of attitude of farmers

Statistics

		How do you rate desires of farmers to spray toxic pesticides on vegetable and khat crop to improve their glossiness?	How do you rate the exposure of consumers to effects of pesticide residues from consumption of food crops supplied by farmers of the study area?	How do you rate the negative effects of pesticide residues that drain into in water bodies from unsafe applications to aquatic life?	How do you rate the negative effects of pesticide residues in soil to organisms living in and on the soil?	how do you rate the overall attitude of communities to use vegetable products harvested from the study area?	how do you rate the overall ecosystems disrupting effects of pesticide residues in the study area?
N	Valid	370	370	370	370	370	370
	Missing	0	0	0	0	0	0

How do you rate desires of farmers to spray toxic pesticides on vegetable and khat crop to improve their glossiness?

		Frequency	Percent	Valid Percent	Cumulative Percent
Valid	very low	51	13.8	13.8	13.8
	low	61	16.5	16.5	30.3

		Frequency	Percent	Valid Percent	Cumulative Percent
	modest	81	21.9	21.9	52.2
	high	120	32.4	32.4	84.6
	very high	57	15.4	15.4	100.0
	Total	370	100.0	100.0	

How do you rate the exposure of consumers to effects of pesticide residues from consumption of food crops supplied by farmers of the study area?

		Frequency	Percent	Valid Percent	Cumulative Percent
Valid	very low	29	7.8	7.8	7.8
	low	52	14.1	14.1	21.9
	modest	112	30.3	30.3	52.2
	high	96	25.9	25.9	78.1
	very high	81	21.9	21.9	100.0
	Total	370	100.0	100.0	

How do you rate the negative effects of pesticide residues that drain into in water bodies from unsafe applications to aquatic life?

		Frequency	Percent	Valid Percent	Cumulative Percent
Valid	very low	32	8.6	8.6	8.6
	low	55	14.9	14.9	23.5
	modest	80	21.6	21.6	45.1
	high	136	36.8	36.8	81.9
	very high	67	18.1	18.1	100.0
	Total	370	100.0	100.0	

How do you rate the negative effects of pesticide residues in soil to organisms living in and on the soil?

		Frequency	Percent	Valid Percent	Cumulative Percent
Valid	very low	20	5.4	5.4	5.4
	low	97	26.2	26.2	31.6
	modest	132	35.7	35.7	67.3
	high	91	24.6	24.6	91.9
	very high	30	8.1	8.1	100.0
	Total	370	100.0	100.0	

How do you rate the overall attitude of communities to use vegetable products harvested from the study area?

		Frequency	Percent	Valid Percent	Cumulative Percent
Valid	very low	39	10.5	10.5	10.5
	low	97	26.2	26.2	36.8
	modest	132	35.7	35.7	72.4
	high	75	20.3	20.3	92.7

		Frequency	Percent	Valid Percent	Cumulative Percent
	very high	27	7.3	7.3	100.0
	Total	370	100.0	100.0	

How do you rate the overall ecosystems disrupting effects of pesticide residues in the study area?

		Frequency	Percent	Valid Percent	Cumulative Percent
Valid	very low	18	4.9	4.9	4.9
	low	95	25.7	25.7	30.5
	modest	116	31.4	31.4	61.9
	high	97	26.2	26.2	88.1
	very high	44	11.9	11.9	100.0
	Total	370	100.0	100.0	

Frequency table of observation and practices of farmers

Statistics

		Do farmers have been using the sale leftover vegetables for their livestock feed?	Do you use all types of vegetables without concern about health effects from pesticides residues?	Do farmers have been mixing their agricultural pesticides near water bodies	Do farmers have been washing pesticide spraying equipment near water bodies	Do farmers use highly toxic chemical pesticides to control crop pests and shine the appearance of some crops?	Do farmers have been applied prohibited pesticides to store crop yields for longer period of time	Do farmers have been supplying vegetables waiting for the standard time gaps in between spraying and supplying	Do farmers have been using vegetables without waiting for the standard time gaps in between spraying and supplying	Do farmers have been using the sale leftover vegetables for their own home consumption?
N	Valid	370	370	370	370	370	370	370	370	370
	Missing	0	0	0	0	0	0	0	0	0

Do farmers have been using the sale leftover vegetables for their livestock feed?

		Frequency	Percent	Valid Percent	Cumulative Percent
Valid	not	150	40.5	40.5	40.5
	yes	220	59.5	59.5	100.0
	Total	370	100.0	100.0	

Do you use all types of vegetables without concern about health effects from pesticides residues?

		Frequency	Percent	Valid Percent	Cumulative Percent
Valid	not	141	38.1	38.1	38.1
	yes	229	61.9	61.9	100.0
	Total	370	100.0	100.0	

Do farmers have been mixing their agricultural pesticides near water bodies

		Frequency	Percent	Valid Percent	Cumulative Percent

		Frequency	Percent	Valid Percent	Cumulative Percent
Valid	not	57	15.4	15.4	15.4
	yes	313	84.6	84.6	100.0
	Total	370	100.0	100.0	

Do farmers have been washing pesticide spraying equipment near water bodies

		Frequency	Percent	Valid Percent	Cumulative Percent
Valid	not	84	22.7	22.7	22.7
	yes	286	77.3	77.3	100.0
	Total	370	100.0	100.0	

Do farmers use highly toxic chemical pesticides to control crop pests and shine the appearance of some crops?

		Frequency	Percent	Valid Percent	Cumulative Percent
Valid	not	204	55.1	55.1	55.1
	yes	166	44.9	44.9	100.0
	Total	370	100.0	100.0	

Do farmers have been applied prohibited pesticides to store crop yields for longer period of time

		Frequency	Percent	Valid Percent	Cumulative Percent
Valid	not	111	30.0	30.0	30.0
	yes	259	70.0	70.0	100.0
	Total	370	100.0	100.0	

Do farmers have been supplying vegetables waiting for the standard time gaps in between spraying and supplying

		Frequency	Percent	Valid Percent	Cumulative Percent
Valid	not	142	38.4	38.4	38.4
	yes	228	61.6	61.6	100.0
	Total	370	100.0	100.0	

Do farmers have been using vegetables without waiting for the standard time gaps in between spraying and supplying

		Frequency	Percent	Valid Percent	Cumulative Percent
Valid	not	86	23.2	23.2	23.2
	yes	284	76.8	76.8	100.0
	Total	370	100.0	100.0	

Do farmers have been using the sale leftover vegetables for their own home consumption?

		Frequency	Percent	Valid Percent	Cumulative Percent
Valid	not	114	30.8	30.8	30.8
	yes	256	69.2	69.2	100.0
	Total	370	100.0	100.0	

DATA FROM SAMPLED EXPERTS

Frequency table of experts' demography

Statistics

		sex of respondents	age of respondents	level of education	years of experience
N	Valid	82	82	82	82
	Missing	0	0	0	0

sex of respondents

		Frequency	Percent	Valid Percent	Cumulative Percent
Valid	male	67	81.7	81.7	81.7
	female	15	18.3	18.3	100.0
	Total	82	100.0	100.0	

age of respondents

		Frequency	Percent	Valid Percent	Cumulative Percent
Valid	<25	2	2.4	2.4	2.4
	26-35	39	47.6	47.6	50.0
	36-45	32	39.0	39.0	89.0
	46-55	8	9.8	9.8	98.8
	>55	1	1.2	1.2	100.0
	Total	82	100.0	100.0	

level of education

		Frequency	Percent	Valid Percent	Cumulative Percent
Valid	diploma	8	9.8	9.8	9.8
	degree and above	74	90.2	90.2	100.0
	Total	82	100.0	100.0	

years of experience

		Frequency	Percent	Valid Percent	Cumulative Percent
Valid	<5	8	9.8	9.8	9.8
	6-10	25	30.5	30.5	40.2
	>10	49	59.8	59.8	100.0
	Total	82	100.0	100.0	

Frequency table of knowledge of experts

Statistics

		Do you know about the negative effects of pesticide residues drained into the water, during the mixing of pesticides and washing sprayer equipment on aquatic life?	Do you know the level of toxicity of pesticides used by farmers for different purposes?	Do you know the negative effects of fishing by pesticide poisoning on aquatic living organisms?	Do you know about the negative effects of pesticide residues in the pesticide-sprayed vegetables and khat crops on consumers?	Do you know the negative effects of pesticide residues in insects killed by pesticides on birds eating the dead insects?	Do you know the poisoning or killing effects of pesticide residues on animals when they feed on sprayed crops or crop by products?	Do you know about the transfer of pesticide residues across food chains/webs?
N	Valid	82	82	82	82	82	82	82
	Missing	0	0	0	0	0	0	0

Do you know about the negative effects of pesticide residues drained into the water, during the mixing of pesticides and washing sprayer equipment on aquatic life?

		Frequency	Percent	Valid Percent	Cumulative Percent
Valid	not	16	19.5	19.5	19.5
	yes	66	80.5	80.5	100.0
	Total	82	100.0	100.0	

Do you know the level of toxicity of pesticides used by farmers for different purposes?

		Frequency	Percent	Valid Percent	Cumulative Percent
Valid	not	17	20.7	20.7	20.7
	yes	65	79.3	79.3	100.0
	Total	82	100.0	100.0	

Do you know the negative effects of fishing by pesticide poisoning on aquatic living organisms?

		Frequency	Percent	Valid Percent	Cumulative Percent
Valid	not	15	18.3	18.3	18.3
	yes	67	81.7	81.7	100.0
	Total	82	100.0	100.0	

Do you know about the negative effects of pesticide residues in the pesticide-sprayed vegetables and khat crops on consumers?

		Frequency	Percent	Valid Percent	Cumulative Percent
Valid	not	12	14.6	14.6	14.6
	yes	70	85.4	85.4	100.0

		Frequency	Percent	Valid Percent	Cumulative Percent
	Total	82	100.0	100.0	

Do you know the negative effects of pesticide residues in insects killed by pesticides on birds eating the dead insects?

		Frequency	Percent	Valid Percent	Cumulative Percent
Valid	not	14	17.1	17.1	17.1
	yes	68	82.9	82.9	100.0
	Total	82	100.0	100.0	

Do you know the poisoning or killing effects of pesticide residues on animals when they feed on sprayed crops or crop by products?

		Frequency	Percent	Valid Percent	Cumulative Percent
Valid	not	6	7.3	7.3	7.3
	yes	76	92.7	92.7	100.0
	Total	82	100.0	100.0	

Do you know about the transfer of pesticide residues across food chains/webs?

		Frequency	Percent	Valid Percent	Cumulative Percent
Valid	not	11	13.4	13.4	13.4
	yes	71	86.6	86.6	100.0
	Total	82	100.0	100.0	

Frequency table of attitude of experts

Statistics

		How do you rate the desires of farmers to spray highly toxic pesticides on vegetable and khat crops to improve the glossiness of leaves?	How do you rate exposures of consumers to the effects of pesticide residues from the consumption of pesticide-sprayed crops supplied by farmers?	How do you rate the negative effects of pesticide residues drained into water bodies from mixing and washing on aquatic life?	How do you rate the negative effects of pesticide residues on soil-inhibiting organisms?	How do you rate your overall interest to use vegetable products harvested from the study area?	How do you rate the overall ecosystems disrupting effects of pesticide residues in the study area?
N	Valid	82	82	82	82	82	82
	Missing	0	0	0	0	0	0

How do you rate the desires of farmers to spray highly toxic pesticides on vegetable and khat crops to improve the glossiness of leaves?

		Frequency	Percent	Valid Percent	Cumulative Percent
Valid	very low	2	2.4	2.4	2.4
	low	8	9.8	9.8	12.2

		Frequency	Percent	Valid Percent	Cumulative Percent
	modest	22	26.8	26.8	39.0
	high	23	28.0	28.0	67.1
	very high	27	32.9	32.9	100.0
	Total	82	100.0	100.0	

How do you rate exposures of consumers to the effects of pesticide residues from the consumption of pesticide-sprayed crops supplied by farmers?

		Frequency	Percent	Valid Percent	Cumulative Percent
Valid	low	3	3.7	3.7	3.7
	modest	15	18.3	18.3	22.0
	high	27	32.9	32.9	54.9
	very high	37	45.1	45.1	100.0
	Total	82	100.0	100.0	

How do you rate the negative effects of pesticide residues drained into water bodies from mixing and washing on aquatic life?

		Frequency	Percent	Valid Percent	Cumulative Percent
Valid	very low	1	1.2	1.2	1.2
	low	6	7.3	7.3	8.5
	modest	10	12.2	12.2	20.7
	high	19	23.2	23.2	43.9
	very high	46	56.1	56.1	100.0
	Total	82	100.0	100.0	

How do you rate the negative effects of pesticide residues on soil-inhibiting organisms?

		Frequency	Percent	Valid Percent	Cumulative Percent
Valid	low	12	14.6	14.6	14.6
	modest	20	24.4	24.4	39.0
	high	26	31.7	31.7	70.7
	very high	24	29.3	29.3	100.0
	Total	82	100.0	100.0	

How do you rate your overall interest to use vegetable products harvested from the study area?

		Frequency	Percent	Valid Percent	Cumulative Percent
Valid	low	20	24.4	24.4	24.4
	modest	24	29.3	29.3	53.7
	high	20	24.4	24.4	78.0
	very high	18	22.0	22.0	100.0
	Total	82	100.0	100.0	

How do you rate the overall ecosystems disrupting effects of pesticide residues in the study area?

		Frequency	Percent	Valid Percent	Cumulative Percent
Valid	very low	1	1.2	1.2	1.2
	low	20	24.4	24.4	25.6
	modest	24	29.3	29.3	54.9
	high	19	23.2	23.2	78.0
	very high	18	22.0	22.0	100.0
	Total	82	100.0	100.0	

Frequency table of observation and practices of farmers and experts

Statistics

		Do farmers mix their agricultural pesticides near water bodies	Do farmers wash pesticide spraying equipment near water bodies	Do farmers use spraying highly toxic chemical pesticides to control crop pests and shine the leaves of some crops?	Do farmers apply prohibited pesticides to store crops for longer period of time?	Do farmers supply vegetables and khat without waiting for the required time interval between the pesticide spraying and the crops supplying?	Do farmers use vegetables without waiting for required time interval between pesticide spraying & crops supplying?	Do farmers use the unsold vegetables for their own home consumption?	Do farmers feed the unsold vegetables for their livestock?	Do you use all types of vegetables without concern about health risks from pesticide residues?
N	Valid	82	82	82	82	82	82	82	82	82
	Missing	0	0	0	0	0	0	0	0	0

Frequency Table

Do farmers mix their agricultural pesticides near water bodies

		Frequency	Percent	Valid Percent	Cumulative Percent
Valid	not	12	14.6	14.6	14.6
	yes	70	85.4	85.4	100.0
	Total	82	100.0	100.0	

Do farmers wash pesticide spraying equipment near water bodies

		Frequency	Percent	Valid Percent	Cumulative Percent
Valid	not	11	13.4	13.4	13.4
	yes	71	86.6	86.6	100.0
	Total	82	100.0	100.0	

Do farmers use spraying highly toxic chemical pesticides to control crop pests and shine the leaves of some crops?

		Frequency	Percent	Valid Percent	Cumulative Percent
Valid	not	28	34.1	34.1	34.1

		Frequency	Percent	Valid Percent	Cumulative Percent
	yes	54	65.9	65.9	100.0
	Total	82	100.0	100.0	

Do farmers apply prohibited pesticides to store crops for longer period of time?

		Frequency	Percent	Valid Percent	Cumulative Percent
Valid	not	22	26.8	26.8	26.8
	yes	60	73.2	73.2	100.0
	Total	82	100.0	100.0	

Do farmers supply vegetables and khat without waiting for the required time interval between the pesticide spraying and the crops supplying?

		Frequency	Percent	Valid Percent	Cumulative Percent
Valid	not	25	30.5	30.5	30.5
	yes	57	69.5	69.5	100.0
	Total	82	100.0	100.0	

Do farmers use vegetables without waiting for required time interval between pesticide spraying & crops supplying?

		Frequency	Percent	Valid Percent	Cumulative Percent
Valid	not	37	45.1	45.1	45.1
	yes	45	54.9	54.9	100.0
	Total	82	100.0	100.0	

Do farmers use the unsold vegetables for their own home consumption?

		Frequency	Percent	Valid Percent	Cumulative Percent
Valid	not	36	43.9	43.9	43.9
	yes	46	56.1	56.1	100.0
	Total	82	100.0	100.0	

Do farmers feed the unsold vegetables for their livestock?

		Frequency	Percent	Valid Percent	Cumulative Percent
Valid	not	48	58.5	58.5	58.5
	yes	34	41.5	41.5	100.0
	Total	82	100.0	100.0	

Do you use all types of vegetables without concern about health risks from pesticide residues?

		Frequency	Percent	Valid Percent	Cumulative Percent
Valid	not	57	69.5	69.5	69.5
	yes	25	30.5	30.5	100.0
	Total	82	100.0	100.0	
